# Casein SNP in Norwegian goats: additive and dominance effects on milk composition and quality

**DOI:** 10.1186/1297-9686-43-31

**Published:** 2011-08-24

**Authors:** Binyam S Dagnachew, Georg Thaller, Sigbjørn Lien, Tormod Ådnøy

**Affiliations:** 1 Department of Animal and Aquacultural Sciences, Norwegian University of Life Sciences, P.O. Box 5003, N-1432 Ås, Norway; 2 Institute of Animal Breeding and Husbandry, Christian-Albrechts University, 24098 Kiel, Germany; 3 Center for Integrative Genetics, Norwegian University of Life Sciences, P.O. Box 5003, N-1432 Ås, Norway

## Abstract

**Background:**

The four casein proteins in goat milk are encoded by four closely linked casein loci (*CSN1S1*, *CSN2*, *CSN1S2 *and *CSN3*) within 250 kb on caprine chromosome 6. A deletion in exon 12 of *CSN1S1*, so far reported only in Norwegian goats, has been found at high frequency (0.73). Such a high frequency is difficult to explain because the national breeding goal selects against the variant's effect.

**Methods:**

In this study, 575 goats were genotyped for 38 Single Nucleotide Polymorphisms (SNP) located within the four casein genes. Milk production records of these goats were obtained from the Norwegian Dairy Goat Control. Test-day mixed models with additive and dominance fixed effects of single SNP were fitted in a model including polygenic effects.

**Results:**

Significant additive effects of single SNP within *CSN1S1 *and *CSN3 *were found for fat % and protein %, milk yield and milk taste. The allele with the deletion showed additive and dominance effects on protein % and fat %, and overdominance effects on milk quantity (kg) and lactose %. At its current frequency, the observed dominance (overdominance) effects of the deletion allele reduced its substitution effect (and additive genetic variance available for selection) in the population substantially.

**Conclusions:**

The selection pressure of conventional breeding on the allele with the deletion is limited due to the observed dominance (overdominance) effects. Inclusion of molecular information in the national breeding scheme will reduce the frequency of this deletion in the population.

## Background

Under normal conditions, the milk of mammals contains 30-35 g of protein per liter [1]. In the milk of ruminants, more than 95% of these proteins are synthesized from six structural genes [2]. The two main whey proteins, α-lactalbumin and β-lactoglobulin, are encoded by the *LALBA *and *LGB *genes, respectively [3]. The four acid-precipitated proteins (caseins) - α_S1_-CN, β-CN, α_S2_-CN and κ-CN - are encoded by four tightly linked casein genes [2]. These four casein loci are found in the following order: *CSN1S1*, *CSN2*, *CSN1S2 *and *CSN3 *within 250 bp on caprine chromosome 6 [2,4-7]. In goats and other ruminants, casein represents about 80% of the total proteins [2].

Casein genetic variants have been identified and characterized in different species (for a review see Ng-Kwai-Hang and Grosclaude [3]). Caroli et al. [8] have reported a comparison among casein genetic variants in cattle, goat and sheep. Analysis of caseins in goats is complex due to extensive polymorphism in the four casein loci [4]. The *CSN1S1 *gene has a 16.5 kb long transcriptional unit composed of 19 exons, which vary in length from 24 bp to 358 bp [9], and 18 introns [5]. So far, more than 16 alleles have been detected and grouped into four classes based on different expression levels of α_S1_-CN in the milk. "Strong" variants (*A*, *B1*, *B2*, *B3*, *B4*, *C*, *H*, *L *and *M*) produce around 3.6 g of α_S1_-CN per liter of milk [10], "medium" variants (*E *and *I*) produce 1.6 g of α_S1_-CN, "weak" alleles (*F *and *G*) produce 0.6 g of α_S1_-CN [2,11] and "null" alleles (*01*, *02*, and *N*) result in absence of the α_S1_-CN fraction in milk [2,4,11,12].

The β-casein, which is encoded by the *CSN2 *locus, is the major casein fraction in goat milk [13]. The *CSN2 *gene consists of nine exons varying in length from 24 bp to 492 bp [2]. Three *CSN2 *genetic variants (A, B and C) are associated with a normal β-CN content [4,14] and two null alleles (0 and 0') result in absence or a reduced level of β-CN [13,15].

Caroli et al. [4] have reviewed the genetic variants of *CSN1S2*; seven variants have been identified among which five are associated with a normal α_S2_-CN level, one with a low level and one resulting in no α_S2_-CN [16]. At the *CSN3 *locus, 15 polymorphic sites have been identified leading to 16 *CSN3 *alleles and 13 κ-casein variants [4,17,18].

Several studies have analyzed the effects of the polymorphism of casein genes on dairy performance and milk quality in different goat breeds [12,19-22]. They have revealed that polymorphisms in the *CSN1S1 *locus have significant effects on casein content, total protein content, fat content and technological properties of milk. It has also been reported that κ-casein (*CSN3*) variants have a significant influence on milk production traits [22,23].

Norwegian dairy goat is a landrace, reared throughout Norway and mainly kept for milk production. In this population, 38-40 Single Nucleotide Polymorphisms (SNP) have been identified within the four casein loci and used in several studies [20]. Most of these polymorphisms are located in the promoter regions of the genes: with 15 SNP in *CSN1S1*, six in *CSN2*, five in *CSN1S2 *and 13 in *CSN3*. A deletion in exon 12 of *CSN1S1*, so far only reported in Norwegian dairy goats, has been found at a high frequency (0.73, [20]). This deletion and a deletion in exon 9, at lower frequency (0.08, [20]) also described in other breeds, are believed to contribute to the unusually high frequency (0.70, [24]) of "null" α_S1_-CN in Norwegian goats milk. Three polymorphisms have been identified at this position of exon 12 and coded as allele 1, 3 and 6 [20], i.e., allele 1: CTG**AAAAA**TAC (deletion), allele 3: CTG**AAGAAA**TAC and allele 6: CTG**AAAAAA**TAC.

Allele 1 is associated with a reduced level of dry matter (DM) content in milk and influences the physico-chemical properties of milk [19,20,24]. The primary goal in the national goat breeding programme is to increase DM production per goat and year, but also to increase the DM content in milk to improve milk quality. In light of this breeding goal, the high frequency of allele 1, which decreases DM yield, is difficult to explain. So far, in this population, only the average production per genotype of the daughters of bucks with known genotypes has been studied [20]. Thus, it has not been possible to identify dominance effects. In this study, milk producing goats were genotyped, and both additive and dominance effects of genes were determined. We investigated the effect of SNP within casein genes on Norwegian goats' dairy performance and milk taste.

## Methods

### Materials

**Genotyping data: **Blood samples were collected from goats of six farms located in southern Norway and genomic DNA was isolated according to standard procedures. Genotyping of 38 SNP was performed with the Sequenom MassARRAY genotyping platform [25] using the assay and genotyping protocols described by Hayes et al. [20]. Identities of the SNP and genotyping conditions are included in additional file 1 (see additional file 1).

Thirty-eight markers - 36 SNP, one deletion, and another position with a deletion or two alternative bases (A or G) - located over the four casein loci were investigated. The deletion and the A or G are named 'SNP14', but have three alleles as explained above. Table 1 presents a summary of the 38 markers (or SNP) used in the study i.e. fourteen SNP in *CSN1S1 *(seven in the promoter, six in the exons, and one in an intron), six SNP in *CSN2 *(five in the promoter and one in an exon), four SNP in *CSN1S2 *(all in exons) and 14 SNP in *CSN3 *(13 in the promoter and one in an exon). The SNP numbering follows Hayes et al. [20].

**Table 1:** Casein genes SNP' position and frequencies in Norwegian dairy goats

**SNP**^**A**^	Gene	Location	**Alleles**^**B**^	**Frequency of rare allele**^**C**^
1	CSN1S1	Promoter	A(G)	0.050
2	CSN1S1	Promoter	C(T)	0.049
4	CSN1S1	Promoter	G(A)	0.130
5	CSN1S1	Promoter	G(A)	0.145
6	CSN1S1	Promoter	G(A)	0.147
7	CSN1S1	Promoter	C(T)	0.146
8	CSN1S1	Promoter	G(A)	0.068
9	CSN1S1	Exon 4	T(C)	0.150
10	CSN1S1	Exon 5	C(G)	0.160
11	CSN1S1	Exon 9	C(D)	0.037
12	CSN1S1	Intron 8	A(G)	0.148
13	CSN1S1	Exon 10	C(G)	0.148
14	CSN1S1	Exon 12	Allele 1 (D)	0.737
			Allele 3 (G)	0.112
			Allele 6 (A)	0.151
15	CSN1S1	Exon 17	C(T)	0.116
16	CSN2	Exon 7	T(C)	0.062
17	CSN2	Promoter	A(G)	0.061
18	CSN2	Promoter	G(A)	0.024
19	CSN2	Promoter	A(G)	0.060
20	CSN2	Promoter	(A)T	0.060
21	CSN2	Promoter	C(T)	0.064
22	CSN1S2	Exon 3	G(A)	0.078
24	CSN1S2	Exon 16	C(G)	0.050
25	CSN1S2	Exon 16	C(T)	0.318
26	CSN1S2	Exon 16	A(T)	0.315
27	CSN3	Promoter	G(A)	0.421
28	CSN3	Promoter	G(A)	0.493
29	CSN3	Promoter	(A)G	0.002
30	CSN3	Promoter	T(A)	0.494
31	CSN3	Promoter	T(A)	0.466
32	CSN3	Promoter	G(C)	0.494
33	CSN3	Promoter	T(G)	0.465
34	CSN3	Promoter	T(G)	0.480
35	CSN3	Promoter	A(G)	0.092
36	CSN3	Promoter	T(C)	0.317
37	CSN3	Promoter	G(T)	0.328
38	CSN3	Promoter	A(G)	0.180
39	CSN3	Promoter	G(A)	0.092
40	CSN3	Exon 4	C(T)	0.098

^A ^Numbering of SNP is according to Hayes et al., 2006 [20]

^B ^The allele in parentheses refers to the minor allele for the SNP. 'D' in SNP11 and SNP14 refer to a deletion.

^C ^For SNP14 frequencies are reported for all the three possible alleles

The extent of the linkage disequilibrium (LD) among these casein SNP was calculated and visualized using the HaploView program [26]. The LD was measured by *r^2 ^*and displayed as shades of grey (the intensity of the grey color relates to the amount of LD between the SNP). Additional information such as the total length of each casein locus and the distances between adjacent casein loci were obtained from literature [5,9] and from the bovine genome [27].

**Production data: **The Norwegian Dairy Goat Control recording system collects data from all flocks participating in milk recording (74.1% of all goat flocks in 2005 [28]), involving both flocks within and outside the buck-circle system [29]. Records from the six farms with genotyped goats were used for this analysis. In each farm, only genotyped goats with kidding date between August 2004 and August 2005 were considered and the phenotypic records correspond to the 2005 production year.

**Daily milk yield (DMY): **refers to the test-day amount of milk in kg as the sum of morning and evening milk production for a single goat. DMY is recorded at least five times per farm per year. For this study, a total of 3194 DMY were available from 575 genotyped goats.

**Milk composition: **includes milk fat content, protein content, and lactose content measured as percent of total milk; somatic cell count (logSCC) and free fatty acids (logFFA) concentration in milk. These measurements are Fourier Transform Infrared (FTIR) spectra based predictions. Among the test-day milk samples, at least three are analyzed for milk content (for either morning or evening milk or both for a test-day). For this study, 2236 milk content measures were available for the 575 genotyped goats.

**Milk taste: **is an organoleptic evaluation of milk taste by dairy personnel on a scale 1 to 4, depending on how much stale/rancid taste the milk has ("besk/harsk" are the Norwegian terms used for the evaluation of milk taste). The scale is defined as 1 - there is no stale/rancid taste, 2 - trace of strong stale/rancid taste, 3 - a stale/rancid taste detected and 4 - stale/rancid taste is strong. For this study, 1352 milk taste scores belonging to 499 genotyped goats were available from five of the six farms.

**Pedigree record: **7325 pedigree records including the 575 genotyped goats were available. The genotyped goats are progenies of 157 bucks. The pedigree file contains full identification of individuals and their parents. A maximum of seven generations back in the pedigree were considered when constructing additive genetic relationship matrix (A).

**Variance components: **the variance components used in the analysis are presented in Table 2. These variance components were obtained from the Norwegian Association of Sheep and Goat Breeders (Norsk Sau og Geit, NSG), which is responsible for running the goat breeding scheme and calculating breeding values. In this study, variance components estimated in January 2009 based on a large dataset were used (unpublished).

**Table 2:** Variance components used for the analysis

	**Traits**^**B**^					
	
**Variance components**^**A**^	Milk yield kg	Fat percentage	Protein percentage	Lactose percentage	log(FFA)	log(SCC)
Additive genetic	0.0532	0.1398	0.0149	0.0133	0.1782	0.0811
Permanent environment	0.0710	0.0629	0.0073	0.0061	0.0979	0.1949
Residual	0.1531	0.3117	0.0196	0.0159	0.2438	0.5157

^A^The variance components were estimated in January 2009 by NSG.

^B^Milk composition traits are expressed in percentage of total milk.

### Data analysis

To separate the effect of single SNP from additive polygenic effects, a mixed model was fitted to our dataset. Two slightly different models were used to analyze different traits.

**Model 1: **a single trait test-day mixed model was used to analyze the individual SNP effect on daily milk production in kg, milk composition traits, somatic cell count (logSCC) and free fatty acid (logFFA). Each SNP effect was fitted as a fixed effect and analysed for one SNP at a time (i.e. the model was run 38 times per trait).

Where:

trait_ijklm_: test-day measure of a trait

μ: fixed effect of the mean

DIM15_i_: fixed effect of stage of lactation, defined in 15-days intervals (*DIM*15*_i_*, where *i = 1,...,24)*.

YS_j_: fixed effect of the kidding season *j (j = 1, 2, 3)*. Three kidding seasons considered: 1- December to February, 2- March to May and 3- June to November

FTD_k_: fixed effect of the farm-test-day *k *(*k *= *1, 2, ..., 34 *for daily milk yield and *k *= *1, 2,...,25 *for milk composition traits)

a_l_: fixed **additive effect **of the major allele of SNP *l *(*l *= *1, 2,...,38*)

d_l_: fixed **dominance effect **of the major allele of SNP *l*

u_m_: random polygenic effects (breeding values) of the animal *m *(*m = 1, 2,...,575)*

p_m_: random permanent environment effect of the animal *m *(*m = 1, 2,...,575)*

e_ijklm_: random residual effect of observation *ijklm*

Matrix representation of the model:

Where: **y **is the vector of phenotypic observations, **X **is a design matrix of fixed effects, other than SNP effects, **Q **is a design matrix of a SNP (additive and dominance) effects, **β **is a vector of fixed non-genetic effects, **q **is a vector of fixed SNP effects (additive and dominant), **Z **is an incidence matrix relating individuals' phenotypes to breeding values **u **and permanent environment effect **p **and **e **is the vector of residual error associated with each observation. The vector of breeding values, **u**, contains only animals with records. Here we assumed ,  and where **A_u _**is subset of the additive genetic relationship matrix (A), which contains only genotyped animals (part of matrix A is used to minimize computation time since the model is run 38 times per trait), **I **is an identity matrix, ,  and are additive genetic, permanent environmental and residual variances, respectively. Q = [Q_a _Q_d_] was set for **additive **and **dominance **effects as follows:

**Model 2: **A slightly different model was used to estimate individual SNP effects on milk taste. Due to fewer observations available for this trait compared to other milk production traits, a longer interval (30 days) was used to account for the effect of stage of lactation (DIM). No polygenic effect was included (because milk taste is not included as a breeding criterion and reliable variance component estimates from a large dataset are not available). To account for genetic relatedness, milk taste scores were corrected for bucks' effects prior to modelling. The correction was done through fitting bucks as a fixed effect in a linear model and collecting the residuals. The residuals of the taste scores were then fitted as in model 2.

The model components were as defined in model 1.

Dominance effects of SNP2, SNP11, SNP18, SNP19, SNP20, SNP24 and SNP29 were not estimated because the number of homozygous goats for the rare alleles of these SNP was either very low or zero. For these SNP, Q_a _was set as 2, 1, and 0 if the SNP is homozygous for the major allele, heterozygous and homozygous for the other allele, respectively.

### Gene substitution effect (α)

Gene substitution effect, *α*, for a SNP is the average change of genotypic value that results when one allele is replaced by the other allele of same locus [30]. Estimated additive (a_l_) and dominance (d_l_) effects of SNP were collected from model 1 and model 2, and gene substitution effects (*α_l_*) were calculated (*α_1 _*= *a_1_*+(1-2 *p_i_*)*d_i _*[31]); where p_l _is the frequency of the major allele at l^th ^SNP position.

### SNP14 genotype's effect

In the analysis of single SNP fixed effects, the three alleles at exon 12 of *CSN1S1 *(SNP14) were first treated as a deletion (allele 1) or a non-deletion (alleles 3 and 6) in both models. In order to quantify the effect of this polymorphism more precisely, the fixed effects of the six possible genotypes ('1/1', '3/3', '6/6', '1/3', '1/6', and '3/6') were also analyzed separately. The effects of these genotypes were also estimated using models 1 and 2, replacing the SNP effect term.

### Statistical inference

To determine the significance of the effect of single SNP, the null hypotheses that there is no additive effect of a SNP (a_l _= 0) and the null hypotheses that there is no dominance effect (d_l _= 0) were tested. The student t-distribution was used to test the significance of each SNP effect on each trait. Due to multiple testing, a Bonferroni threshold correction was applied to obtain a 5% overall error rate when testing for the 38 SNP per trait. The effective number of independent tests was determined using a method that takes the linkage disequilibrium (LD) structure into account as described in Cheverud (2001) [32]. If the dominance effect (d_l_) of a SNP was significant, the degree of dominance (k_l _= d_l_/a_l_) was determined for the SNP. If k_l _was greater than 1, the significance of the overdominance effect was checked by testing *H*_1_: *d_l_-a_l _*> 0. Also, the null hypotheses that there is no difference between the *CSN1S1 *genotype '1/1' (homozygous for deletion) and each of the other five exon 12 *CSN1S1 *genotypes were tested.

### Statistical tools

Scripts were written for each model and run in **'R' **statistical software (R Development Core Team) [33].

## Results

### Linkage disequilibrium (LD) structure

**Figure 1:**
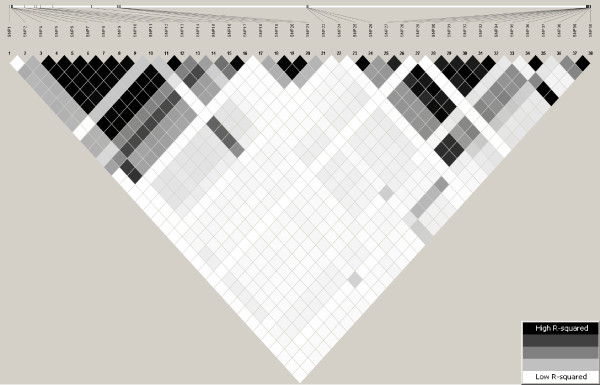
**Graphical representation of Linkage Disequilibrium (LD) across SNP within four casein loci in Norwegian dairy goats**. Each diamond indicates the extent of pairwise LD measured by r^2 ^between the SNP specified; the darker the color, the higher the r^2 ^value (white, r^2 ^= 0; shades of grey, 0 < r^2 ^< 1 and black, r^2 ^= 1); the r^2 ^values used to generate this graphical representation are given in additional file 2 (see additional file 2)

Figure 1 is a graphical representation of the extent and distribution of LD within the four casein loci in Norwegian dairy goats. Pairwise LD values used to create the figure are given in additional file 2 (see additional file 2). Figure 1 includes *CSN1S1 *SNP 1-14, *CSN2 *SNP 15-20, *CSN1S2 *SNP 21-24 and *CSN3 *SNP 25-38. A substantial amount of LD was observed among the casein SNP. The observed LD varied from completely linked (r^2 ^= 1, black) to no LD (r^2 ^= 0, white). Figure 1 shows that *CSN2 *SNP are in stronger LD with *CSN1S1 *SNP than they are with SNP of the *CSN1S2 *and *CSN3 *genes. It also shows the *CSN1S2 *SNP are in strong LD with *CSN3 *SNP.

### Test of SNP effects

**Figure 2:**
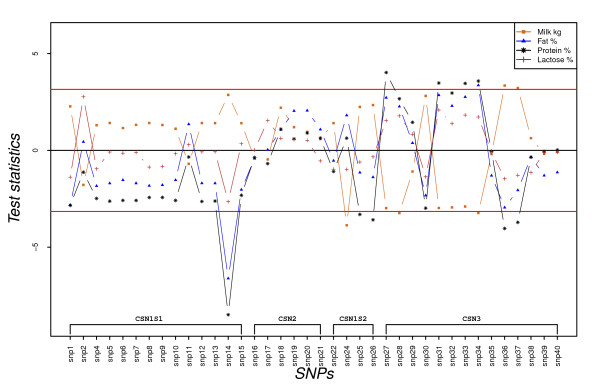
**SNP's additive effect on milk production in kg, protein %, fat % and lactose % expressed as test statistics for frequent alleles**. Test statistics (estimated effects divided by their standard errors) are embedded in the y-axis; the horizontal lines indicate 5% experiment-wise level of significance and any SNP having a test statistic value for a trait above the top line or below the bottom line indicates that it has a significant effect on the trait.

**Figure 3:**
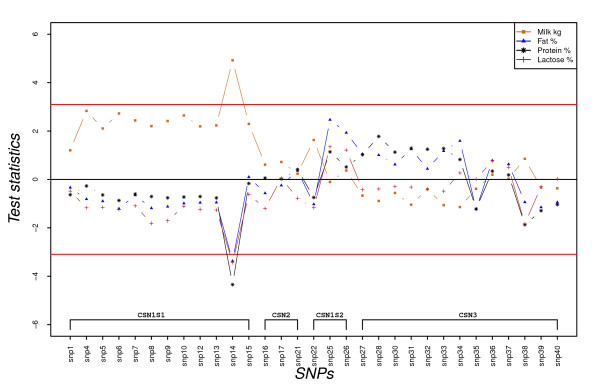
**SNP's dominance effect on milk production in kg, protein %, fat % and lactose % expressed as test statistics for frequent alleles**. Test statistics (estimated effects divided by their standard errors) are embedded in the y-axis; the horizontal lines indicate 5% experiment-wise level of significance and any SNP having a test statistic value for a trait above the top line or below the bottom line indicates that it has a significant effect on the trait.

**Figure 4:**
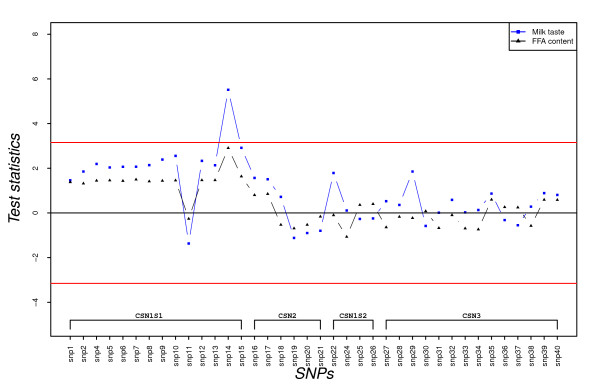
**SNP's additive effect on milk taste and FFA concentration in milk expressed as test statistics for frequent alleles**. Test statistics (estimated effects divided by their standard errors) are embedded in the y-axis; the horizontal lines indicate 5% experiment-wise level of significance and any SNP having a test statistic value for a trait above the top line or below the bottom line indicates that it has a significant effect on the trait.

The test statistics of estimates for the major alleles at each SNP position are plotted in Figures 2, 3 and 4. Figures 2 and 3 present t-statistics values for additive (a_l_) and dominance (d_l_) effects of single SNP_l _on milk production traits. Individual SNP show a similar pattern of additive effects for protein and fat content in milk (Figure 2). At most positions, the observed t-statistics for protein percentage are higher than for fat percentage. Among the SNP within *CSN1S1*, only SNP14 deletion (allele 1) significantly reduces both fat and protein percentages at the chosen error rate. Two SNP within *CSN1S2 *(SNP25 and SNP26) had significant negative effects for protein percentage with an opposite trend for milk production in kg. The major allele of *CSN1S2 *SNP24 was associated with a significantly lower milk yield at the chosen error rate (Figure 2).

A cluster of SNP at *CSN3 *(SNP27-SNP29 and SNP31-SNP34) had a tendency to increase protein % and fat % and to reduce milk production in kg. However, few of these SNP had significant additive effects: SNP28, SNP34, SNP36 and SNP37 for milk production in kg, SNP27, SNP31, SNP33, SNP34, SNP36 and SNP 37 for protein % and SNP34 for fat % (Figure 2). Almost all the SNP within *CSN1S1 *and *CSN3 *loci had opposite additive effects on milk yield and milk content traits. The deletion in exon 9 of *CSN1S1 *(SNP11), which results in the absence of detectable α_S1_-casein [12], did not show any significant additive effect, but also did not follow the pattern of the neighbouring SNP.

The dominance effects of casein SNP for milk production in kg, protein %, fat %, and lactose % are presented in Figure 3. As for additive effects of these SNP, similar patterns of dominance effects was observed for protein % and fat %. Only the deletion in exon 12 of *CSN1S1 *(SNP14) had significant dominance effects for milk production in kg and milk composition (the heterozygote at this position had significantly higher milk production in kg, and lower protein %, fat %, and lactose % than the average values of the homozygotes). As for the additive effects, all SNP in the *CSN1S1 *locus had opposite dominance effects on milk yield and milk composition traits (Figure 3).

For the traits with significant dominance, the degrees of dominance are presented in Table 3. The ratios are between 0.5 and 1, indicating partial dominance, for protein % and fat % and higher than 1, implying overdominance, for milk production in kg and lactose %. The overdominance effects of SNP14 are significant (*p < 0.01*) for milk production in kg and weakly significant (*p < 0.1*) for lactose % (Table 3).

**Table 3:** SNP14 additive, dominance effects and dominance to additive ratio for milk production traits.

Traits	Effects		Degree of dominance [k = d/a]	**P-values**^**A**^
			
	Additive [a]	Dominance [d]		
Milk yield (kg)	0.0932	0.2016	2.16	0.0011
Lactose (%)	-0.0327	-0.0538	1.65	0.064
Fat (%)	-0.2890	-0.1698	0.59	-
Protein (%)	-0.1136	-0.0736	0.65	-

^A ^P-values are for testing if the difference between d and a is significantly greater than zero.

Single SNP fixed additive effects on milk taste and free fatty acid (logFFA) concentration in milk are presented in Figure 4. Additive effects of casein SNP on milk taste follow a pattern similar to that of FFA concentration in milk (Figure 4). The deletion in exon 12 of *CSN1S1 *(SNP14) showed a significant additive effect on milk taste - i.e. was associated with a stronger rancid/stale taste - at the chosen level of significance. However, none of the SNP had significant additive effects on FFA concentration in milk (Figure 4). No significant dominance effects on either of these traits were found (results not presented).

### Gene substitution effect and variance

**Figure 5:**
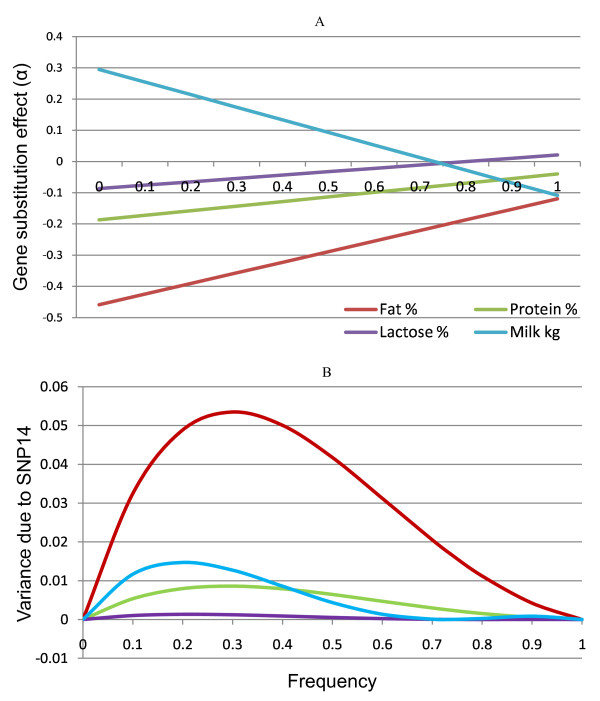
**Gene substitution effect and variance of the SNP14**. Gene substitution effects of SNP14 on milk yield in kg, protein %, fat % and lactose %. The effects are plotted against the frequency of allele 1; the substitution effects are given in kg or % according to the traits. A) Variances due to SNP14 for milk yield in kg, protein %, fat % and lactose %; the variances are plotted against the frequency of allele 1 of SNP14

Figure 5A presents the gene substitution effect (*α*) of SNP14 for the estimated additive (a) and dominance (d) values depending on the different allele 1 (deletion) frequencies. Results of the other SNP are not presented here. Figure 5A shows that the gene substitution effect of the SNP decreases when the frequency of allele 1 increases for milk yield, and becomes negative for allele frequencies above 0.74. For lactose %, the substitution effect would be zero if the frequency of allele 1 were 0.87 and positive for higher frequencies (Figure 5A). The magnitude of the gene substitution effect is also reduced for protein % and fat %, becoming less negative with an increasing frequency of allele 1, but remaining negative (Figure 5A).

The contribution of the gene substitution effect of SNP14 to the additive genetic variance is presented in Figure 5B. This Figure shows that the variance increases for fat % and protein %, reaches maximum and then decreases as the frequency of allele 1(deletion) increases. For milk production in kg and lactose % a similar trend of variance is observed, but after reaching zero at 0.74 for milk and 0.87 for lactose there is a small additive variance contribution for higher allele 1 frequencies. The variances reach their maximum values at frequencies for the allele 1 below 0.5 differing somewhat for the four traits (Figure 5B). The maximum variance contribution of SNP14 might attain approximately half the additive genetic variance given in Table 2 for protein and fat percentages, and less for lactose percentage and milk yield in kg.

### Effect of the genotypes at SNP14

**Figure 6:**
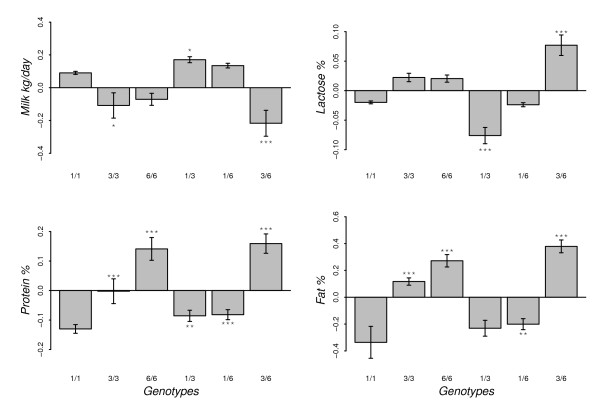
**Effect of SNP14 genotypes on milk yield in kg, lactose %, fat % and protein %**. The bars indicate ± SE, and asterisks indicate a significant difference from genotype homozygous for the deletion ['1/1'] (***, p < 0.01; **, p < 0.05; *, p < 0.1)

**Figure 7:**
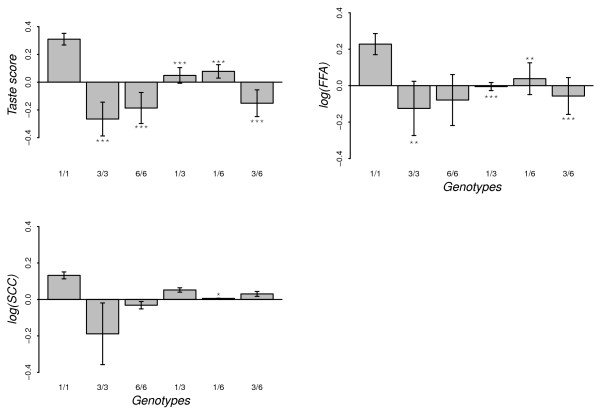
**Effect of SNP14 genotypes on milk taste, SCC, FFA concentration in milk**. The bars indicate ± SE, and asterisks indicate a significant difference from genotype homozygous for the deletion ['1/1'] (***, p < 0.01; **, p < 0.05; *, p < 0.1)

The estimated effects of the six genotypes at exon12 of *CSN1S1 *(SNP14) and the significance tests to compare the differences between the five genotypes and the homozygous genotype for allele 1 ('1/1') are presented in Figures 6 and 7. Figure 6 shows that '3/6' goats produced less milk production in kg (*p < 0.01*) and more lactose (*p < 0.01*) than '1/1' goats. '1/3' goats had a lower lactose % (*p < 0.01*) compared to '1/1' goats. All five genotypes were associated with a significantly higher protein % in milk than that in '1/1' goats. Goats homozygous for allele 1 also had a lower milk fat % compared to '3/3', '6/6', '1/6' and '3/6' (Figure 6).

All the five genotypes - '3/3', '6/6', '1/3', '1/6', and '3/6' - were significantly associated with less strong milk taste compared to genotype homozygous for the deletion (Figure 7). This Figure also shows that the '1/1' genotype led to a significantly higher FFA concentration in the milk in contrast with '3/3','1/3','1/6' and '3/6' genotypes. In addition, although the '1/1' goats had the highest somatic cell count (logSCC), the difference was only weakly significant for the '1/6' genotype (*p < 0.1*, Figure 7)

## Discussion

The effects of casein polymorphisms on dairy performance of different goat breeds have been reviewed across countries [12,18-20]. A previous study on Norwegian goats [20] reported on an association analysis between the casein genotypes of bucks and the daughters' yield deviation (DYD). In this study, both genotype and phenotype information of milk producing goats was used to investigate casein SNP dominance effects in addition to their additive effects. Unlike in the aforementioned study [20], we identified single SNP of *CSN1S1 *and *CSN3 *genes significantly associated with milk production in kg and milk contents (Figure 2) and a SNP in the *CSN1S1 *gene that was significantly associated with milk taste (Figure 4).

One explanation for the higher significance revealed in our study, could be that family analysis in a segregating population cannot disentangle the fixed additive and dominance effects and thus only gene substitution effects could be studied [31]. The substitution effect analysis of SNP14 (Figure 5A) showed that allele 1 had low allele substitution effects on milk and milk composition traits at its current frequency in the population. This contributes to the small effect found in the previous dataset [20].

Effects of *CSN1S1 *polymorphism on milk fat content have been reported in several goat populations [3,12]. To explain this unexpected effect, rather than a direct genetic cause, it is hypothesised that the absence of α_S1_-casein disrupts the intercellular transport of caseins, which in turn disturbs the secretion of milk lipids [34,35]. Our observation on the allele with a deletion in exon 12 of *CSN1S1*, which probably leads to "null" α_S1_-casein, is associated with a reduced fat content of milk (Figure 2 and 6), is in line with this hypothesis.

Hayes et al. [20] have proposed that the observed higher SNP effects at *CSN3 *locus might not be due to direct genetic effects, but rather to the fact that the SNP are physically associated with the causative mutation responsible for the observed variation. However, data reported in other breeds strongly confirmed the effect of κ-casein polymorphisms on milk production traits [22,23,36]. The observed additive effects of *CSN3 *SNP on protein percentage and milk yield (Figure 2) in this study are in agreement with those findings.

The single SNP analyses did not detect any significant associations between casein SNP and FFA concentration in milk (Figure 4). However, when analyzing separately the six genotypes at SNP14 position, a significant variation in FFA concentration was observed (Figure 7). Ådnøy et al. [19] have also reported significant association between *CSN1S1 *genotypes and FFA concentration in milk in goats from two flocks of the same Norwegian breed. FFA are released into the milk through the action of lipase on fat molecules leading to lipolysis [37] and this lipolytic activity may affect negatively the sensory quality of the milk and its products [38] because of the unpleasant flavor produced during this process. Even though several other factors contribute to the taste of goat milk [18], genetic variants at SNP14 position could explain part of the significant variations in milk taste (Figure 4 and 7). This might be related with the FFA concentration in the milk. The results show that genotypes associated with a high concentration of FFA in milk are also associated with a strong milk taste (Figure 7). It has been suggested [21] that milk from goats with "weak" *CSN1S1 *alleles have higher post-milking lipolytic activity than milk from goats with the "strong" *CSN1S1 *alleles. In our study, the "weak" alleles (genotype homozygous for allele 1) tend to be associated with a higher FFA concentration in milk (Figure 7) and support the suggestion.

For SNP14, dominance effect (d) was significantly greater than additivity (a) for milk yield in kg and lactose % (Table 3), implying an overdominance effect for these traits. Based on the estimated a and d, the genetic variances of SNP14 are small at the existing gene frequency (0.73) for milk production in kg, fat, protein and lactose % (Figure 5B). Lynch and Walsh [30] have described that in case of overdominance, there is always an intermediate allele frequency at which genetic variance is equal to zero. Figure 5B shows that the genetic variance of SNP14 is zero at allele frequencies of 0.74 and 0.87 for milk production in kg and lactose %, respectively. The variances became zero (Figure 5B) when the respective gene substitution effects cross the x-axis (Figure 5A).

A primary breeding goal of Norwegian dairy goat population is towards high DM production of milk per goat and year at least since 1996. Nevertheless, the frequency of the deletion in exon12 of *CSN1S1 *gene has remained high (0.73, Table 1) despite the negative effects of the allele on DM content of the milk and milk quality [19,20,24]. Our results also confirmed that allele 1 of SNP14 is associated with significantly reduced protein and fat percentages (Figure 2 and 6).

In practice, breeding sire evaluations are based on their daughters' performance and therefore use only the gene substitution effect variance [31]. If a gene has an additive effect only, the gene substitution effect is equal to the additive effect of the gene. With dominance, the gene substitution effect is no longer equal to the additive effect, but includes a function of the dominance effect and the frequency of the gene in the population [30]. Allele 1 of SNP14 has shown a marked dominance effect on protein % and fat % (Figure 3) and exhibits overdominance for lactose % and milk yield (Table 3). Figure 5A shows that the gene substitution effect (α) of the allele is reduced for milk production in kg when the allele frequency increases until 0.74. It also shows that the magnitude of the gene substitution effect decreases for milk contents when the allele frequency increases. With the current frequency of the allele in the population, 0.73, the gene substitution effect is almost zero for milk production in kg and close to zero for lactose % and the magnitude of the effect is reduced for protein % and fat %. Similarly, Figure 5B shows that the variances of the gene substitution effects are reduced at the higher frequencies of allele 1.

Traditional selection based on gene substitution effect has a low or no pressure on a major gene segregating in a population where the major gene exhibits non-additive variation and the favorable allele is found at a low frequency - as explained by Dodds et al. [39]. In the case of allele 1 of SNP14, the observed dominance effects reduce the gene substitution effects and their variances (additive genetic variances available for selection) for the traits included in the breeding goal. This suggests that the selection pressure of conventional breeding on the allele is limited at the current frequency of allele 1. This could be one explanation why the allele frequency has remained high in spite of the fact that selection is directed against the additive effect of the allele for milk content.

In this study, single SNP effects are found in separate models, modelling one SNP at a time. This would be adequate if the SNP were independent (in linkage equilibrium). The fact that the four casein genes are found clustered within 250 kb implies that they have high tendency of being in high LD (inherited together as haplotypes). Figure 1 shows a considerable amount of LD among casein SNP especially at either end of the chromosome segment containing the casein genes. The result suggests that using haplotypes (or multivariate analysis techniques) to account for the observed LD could be beneficial in association studies as well as in breeding. Moreover, the advantage of using casein haplotypes is that it takes into account not only casein variants but also other important polymorphisms within the casein cluster region (for a review look at Caroli et al. [8]).

## Conclusions

We have shown that the deletion in exon12 of *CSN1S1 *found in Norwegian dairy goats is significantly associated with milk quantity and quality, including milk taste. The allele showed overdominance effects for milk yield in kg and lactose percentage and dominance effects for protein and fat percentages. The observed non-additive effect of the allele with the deletion and its high frequency in the population, 0.73, will reduce the additive genetic variances of the locus available for selection. This limits the selection pressure of conventional breeding on the allele. Use of molecular information in the national breeding scheme would help reduce the frequency of the allele with the deletion in the population (currently, information about the deletions in exon 9 and exon 12 of *CSN1S 1 *is used for the genetic evaluation).

## Competing interests

The authors declare that they have no competing interests.

## Authors' contributions

BD carried out the analysis, and drafted the manuscript. GT participated in supervising the study and editing the manuscript. SL was responsible for genotyping and quality filtering of SNP data and editing the manuscript. TÅ organized and facilitated the research, supervised the study, and finalized the manuscript. All authors read and approved the final manuscript.

## Supplementary Material

Additional file 1 **SNP and genotyping condition**. The file contains identity of 38 SNP used in the study and assay for the genotyping. Click here for file

Additional file 2 **Pairwise linkage disequilibrium (LD) among SNP within the four casein loci in Norwegian dairy goats**. The file contains pairwise LD measurements in D' and r^2^. The r^2 ^values are used to generate the graphical representation of LD (Figure 1). Click here for file
